# The DNA damage response is required for oocyte cyst breakdown and follicle formation in mice

**DOI:** 10.1371/journal.pgen.1009067

**Published:** 2020-11-18

**Authors:** Ana Martínez-Marchal, Yan Huang, Maria Teresa Guillot-Ferriols, Mònica Ferrer-Roda, Anna Guixé, Montserrat Garcia-Caldés, Ignasi Roig

**Affiliations:** 1 Unitat de Citologia i Histologia, Departament de Biologia Cel·lular, Fisiologia i Immunologia, Facultat de Biociències, Universitat Autònoma de Barcelona, Cerdanyola del Vallès, Spain; 2 Grup d’Inestabilitat i Integritat del genoma, Institut de Biotecnologia i Biomedicina, Universitat Autònoma de Barcelona, Cerdanyola del Vallès, Spain; 3 Unitat de Biologia Cel·lular i Genètica Mèdica, Facultat de Medicina, Universitat Autònoma de Barcelona, Cerdanyola del Vallès, Spain; Harvard Medical School, UNITED STATES

## Abstract

Mammalian oogonia proliferate without completing cytokinesis, forming cysts. Within these, oocytes differentiate and initiate meiosis, promoting double-strand break (DSBs) formation, which are repaired by homologous recombination (HR) causing the pairing and synapsis of the homologs. Errors in these processes activate checkpoint mechanisms, leading to apoptosis. At the end of prophase I, in contrast with what is observed in spermatocytes, oocytes accumulate unrepaired DSBs. Simultaneously to the cyst breakdown, there is a massive oocyte death, which has been proposed to be necessary to enable the individualization of the oocytes to form follicles. Based upon all the above-mentioned information, we hypothesize that the apparently inefficient HR occurring in the oocytes may be a requirement to first eliminate most of the oocytes and enable cyst breakdown and follicle formation. To test this idea, we compared perinatal ovaries from control and mutant mice for the effector kinase of the DNA Damage Response (DDR), CHK2. We found that CHK2 is required to eliminate ~50% of the fetal oocyte population. Nevertheless, the number of oocytes and follicles found in *Chk2-*mutant ovaries three days after birth was equivalent to that of the controls. These data revealed the existence of another mechanism capable of eliminating oocytes. *In vitro* inhibition of CHK1 rescued the oocyte number in *Chk2*^*-/-*^ mice, implying that CHK1 regulates postnatal oocyte death. Moreover, we found that CHK1 and CHK2 functions are required for the timely breakdown of the cyst and to form follicles. Thus, we uncovered a novel CHK1 function in regulating the oocyte population in mice. Based upon these data, we propose that the CHK1- and CHK2-dependent DDR controls the number of oocytes and is required to properly break down oocyte cysts and form follicles in mammals.

## Author summary

In mammals, female fertility is limited by the number of germ cells, i.e., the oocytes, females are born with. These oocytes associate with somatic cells to produce follicles that guarantee the production of a fertilizable egg and the synthesis of sex hormones. During fetal development, oocytes initiate meiosis to halve their ploidy. As part of the meiotic program, oocytes self-induce hundreds of DSBs into their genome, which have to be repaired. However, oocytes are not very efficient at repairing DSBs. Also, during fetal development, a great part of the oocytes are eliminated, in a process that has been linked to the formation of follicles. It has been described that the effector kinase of the DDR (CHK2) is responsible for eliminating oocytes with persistent DSBs. Thus, we hypothesized that the DDR may be responsible for eliminating oocytes. In agreement with our hypothesis, we found that CHK2 was responsible for eliminating most fetal oocytes and regulating follicle formation in the mouse. Unexpectedly, we found that there is a CHK1-dependent perinatal oocyte death occurring in *Chk2*^*-/-*^ mice that alters follicle formation. These data suggest that the CHK2- and CHK1-dependent DDR control the oocyte pool and follicle formation in mammals.

## Introduction

In mice oogenesis, the primordial germ cells migrate to the genital ridges around 10.5 days post-coitus (dpc)[1], become oogonia and proliferate without completing cytokinesis, resulting in the formation of cysts, i.e., groups of cells joined by the cytoplasm [2–4]. Within these cysts, oocytes differentiate and initiate meiosis around 13.5 dpc. But meiotic progression stops at the end of the first meiotic prophase, around birth [5]. Concomitantly, the majority of the oocytes die and cysts break down, so single oocytes can be surrounded by stromal ovarian cells to form primordial follicles [6]. These follicles containing arrested oocytes represent the pool of germ cells females will use during their entire reproductive lifetime.

The massive oocyte elimination occurring during the early stages of oogenesis is not specific to mice, but is common in many other mammalian species [7]. For instance, in humans, only ~10% of the oocytes that initiate meiosis will end up forming a follicle [8]. The reasons behind the effort of eliminating the vast majority of the oocytes produced are still unknown, although it has been speculated that it may help in disassembling cysts to individualize oocytes so follicles can form [9,10]. Additionally, the possible triggers of this massive oocyte death are not completely understood. The activation of the retrotransposable LINE-1 element during fetal development has been shown to trigger oocyte death [11], however, the mechanism behind it is also unknown [12]. Nonetheless, quality-control mechanisms that regulate meiotic prophase progression have been speculated to play a part in this process [7].

Meiosis is the reductional division of the genome that generates haploid cells. At the onset of the first meiotic prophase, SPO11 creates hundreds of double-stranded breaks (DSBs) on the genome, which are repaired by homologous recombination leading to the pairing and synapsis of the homologous chromosomes [13]. These processes are tightly regulated to avoid possible deleterious effects originated by errors in recombination or synapsis. Therefore, when errors occur, meiocytes delay their cell-cycle progression, and can even activate programmed cell death [14]. Two mechanisms have been identified that are able to lead mammalian meiocytes into apoptosis. One dependent on DSB formation and recombination progression, and a second one independent of SPO11-generated DSBs [15–19]. Studies in spermatocytes and oocytes have revealed the central role that the DNA damage-response (DDR) effector kinase, CHK2, has in the arrest of mammalian meiocytes with persistent recombination intermediates [17,19].

The DDR is a complex signaling network that is responsible for the maintenance of genome integrity. Different DNA lesions activate different DDR pathways. Whereas single-stranded DNA breaks activate the Ataxia Telangiectasia and Rad3-related protein (ATR), which in turn activates the effector kinase CHK1, DSBs activate the ataxia telangiectasia mutated (ATM) kinase, which ultimately activates the effector kinase CHK2. Nonetheless, the activation of either of these two DDR pathways promotes the repair of DNA damage or the elimination of the cells which accumulate excessive damage in order to maintain genome stability [20]. Several studies have revealed the role of these two pathways in regulating meiotic recombination in mice [17,21–24].

Mammalian gametogenesis is characterized by a marked sexual dimorphism [25]. One of many examples is found in the distinct control of meiotic recombination that occurs in mammalian oocytes, as compared with spermatocytes [22,26–28]. While spermatocytes repair most DSBs by mid-pachynema, the vast majority of human oocytes still present multiple unrepaired DSBs at the same stage. Based upon the persistence of unrepaired DSBs present in wild-type oocytes at the end of meiotic prophase, we hypothesized that the physiological oocyte death occurring during oogenesis is due to the activation of the CHK2-dependent DDR in those oocytes that accumulate more unrepaired DSBs. Furthermore, due to the co-occurrence of this massive oocyte death and cyst breakdown, we have also hypothesized that the DDR-dependent oocyte elimination contributes to proper cyst breakdown and follicle formation. To test these ideas, the number of oocytes and the cyst breakdown and follicle formation in perinatal female ovaries from control and *Chk2*^-/-^ mice was analyzed. We found that during fetal development, the oocytes with higher amounts of unrepaired DSBs were eliminated in a CHK2-dependent manner, suggesting a selection process of the fittest oocytes to survive fetal development. We also showed that CHK2 is required to eliminate fetal oocytes as a result of LINE-1 activation, suggesting that the mechanism behind LINE-1-triggered oocyte death depends on DDR. Nevertheless, control and *Chk2* mutant samples had the same number of oocytes by 4 days postpartum (dpp). Our results also provide evidence that the CHK1 function is required to eliminate *Chk2*^*-/-*^ oocytes after birth. Moreover, cyst breakdown and follicle formation were also disrupted when the two effector kinases of DDR were inhibited, proving that the regulation of the oocyte population mediated by DDR is required for proper cyst breakdown and follicle formation in mammals.

## Results

### *Chk2* mutant fetal ovaries accumulate oocytes with more unrepaired DSBs than do controls

**Fig 1:**
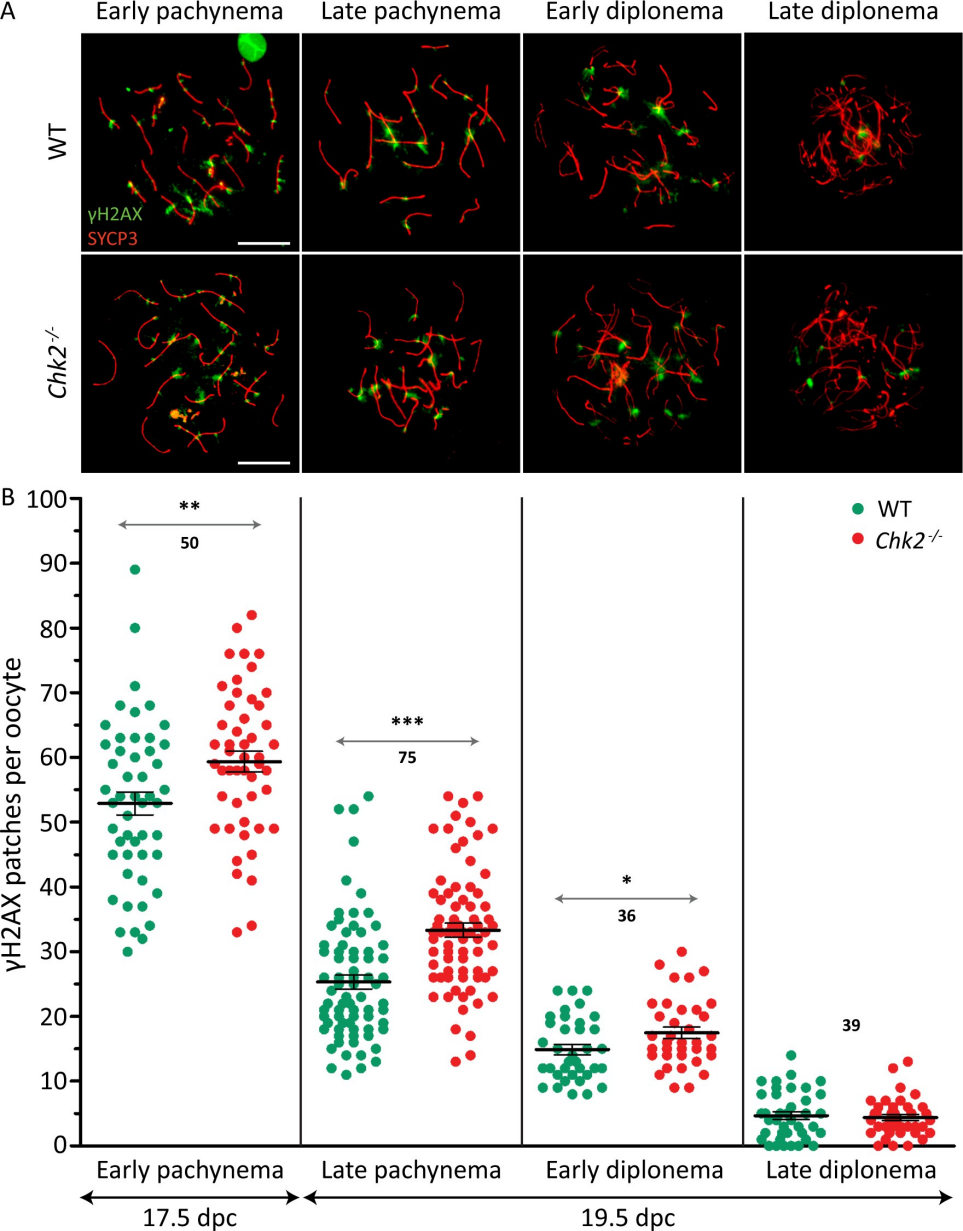
*Chk2*^*-/-*^ oocytes present a higher number of unrepaired DSBs at pachynema and early diplonema than do controls. (A) Representative images of control (WT) and *Chk2*^*-/-*^ oocytes at pachynema and diplonema. The cells are immunostained against SYCP3 (red) and ɣH2AX (green). The scale bars represent 10 μm and applies to all images. (B) Quantification of the number of γH2AX patches found in WT and *Chk2*^*-/-*^ oocytes at pachynema and diplonema. The horizontal lines represent the mean ± standard error of the mean (SEM). *0.05<p<0.01, **0.01<p<0.001, ***p≤0.001 (Unpaired T-test). Data were derived from at least two different animals per condition, and the number of cells counted per each genotype and condition is indicated above.

Our model predicts that in the absence of CHK2, ovaries will accumulate oocytes with high numbers of unrepaired DSBs that normally would be eliminated. To test this, the number of γH2AX patches was counted, as a surrogate of unrepaired DSBs [26], in control and *Chk2* mutant oocytes at different stages of meiotic prophase. As mentioned earlier and described elsewhere [26,28], wild-type oocytes at pachynema presented multiple unrepaired DSBs. Importantly, *Chk2* mutant pachytene-stage oocytes had significantly more patches of γH2AX than did control cells (p<0.0001 T-test, Fig 1, S1 Table and S1 Data). This finding is not due to a delay in the meiotic prophase progression of *Chk2* mutant samples (S1 Fig). Thus, these data corroborate our model and suggest that CHK2 eliminates the oocytes with a higher number of unrepaired DSBs at pachynema. Nonetheless, as oocytes completed meiotic prophase, the difference of unrepaired DSBs between the two genotypes decreased. So that, at late-diplonema, mutant and control oocytes displayed similar numbers of γH2AX patches (p = 0.6856 T-test, Fig 1, S1 Table and S1 Data). This result could be a manifestation of the oocytes’ ability to repair the damage past the pachytene stage, as has been proposed before [19,29]. Alternatively, it could also be interpreted as oocytes which accumulate unrepaired DNA damage were being eliminated by a CHK2-independent mechanism occurring after the pachytene stage.

### Perinatal *Chk2* mutant ovaries have more oocytes than do control ovaries

**Fig 2:**
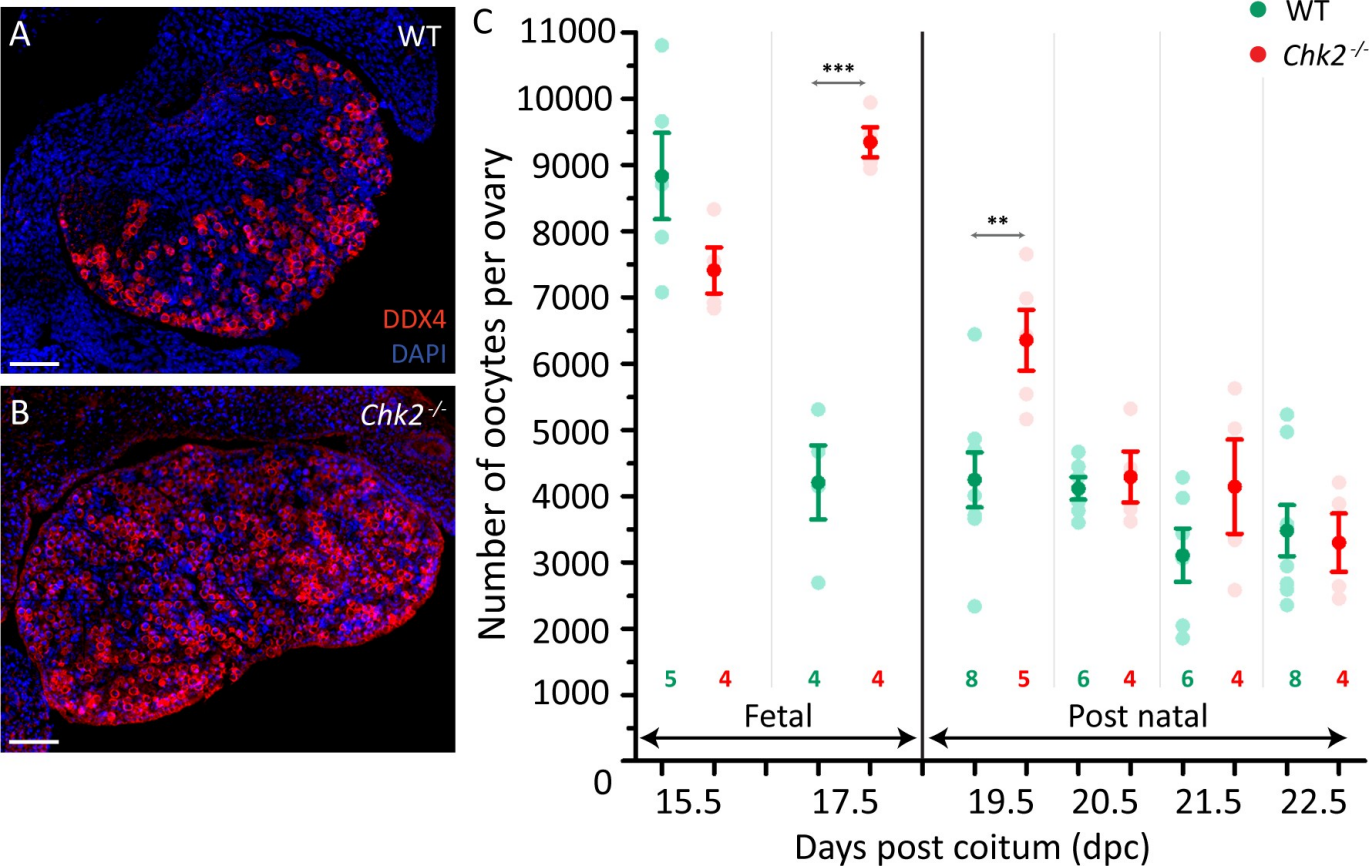
CHK2 determines the number of oocytes in fetal ovaries. (A-B) Representative images of 17.5 dpc control (WT, A) and *Chk2*^*-/-*^ (B) histological sections of ovaries immunostained against DDX4 (red) and the DNA counterstained with DAPI (blue). The scale bar represents 40 μm. (C) Number of oocytes in fetal (15.5–17.5 dpc) and postnatal (19.5–22.5 dpc) control (WT) and *Chk2*^*-/-*^ ovaries. The round symbols represent the mean, and the lines the SEM. Pale circles show the number of oocytes found in each control and *Chk2*^*-/-*^ ovary counted. *0.05<p<0.01, **0.01<p<0.001, ***p≤0.001 (Unpaired T-test). Data were derived from at least two different animals per condition, and the number of ovaries counted per each genotype and condition is indicated below.

Our model also predicts that *Chk2* mutant ovaries should contain more oocytes than wild-type controls. Thus, we counted the number of oocytes present in control and *Chk2* mutant ovaries from 15.5 dpc to 4 dpp (22.5 dpc) mice. We expected that *Chk2* mutant ovaries would present more oocytes than would wild-type samples after most oocytes have reached the pachytene stage, around 17.5–19.5 dpc. To perform this analysis, we serially sectioned the ovaries and immunostained every other section against the DDX4 germ-cell marker (Fig 2A and 2B). Our control mice showed the expected pattern: at 15.5 dpc, ovaries contained the maximum number of germ cells, on average almost 9,000 oocytes (Fig 2C, S2 Table and S1 Data). At 17.5 dpc, half of the control oocytes had been eliminated and the ovaries displayed an average number of 4,207 oocytes. From this stage, the number of oocytes found in our control mice tended to slowly decrease until 4 dpp. As expected, *Chk2* mutant ovaries displayed a similar number of oocytes as did control samples at 15.5 dpc (p = 0.1181, T-test, Fig 2C, S2 Table and S1 Data). However, the number of oocytes found at 17.5 dpc in *Chk2* mutant samples was significantly higher than was the one found in control ones (p = 0.0001, T test). These data suggest that CHK2 is required to physiologically eliminate the fetal oocytes around 16.5 dpc, as our model predicted. Unexpectedly, the number of oocytes present in *Chk2* mutant ovaries declined significantly during the following days, until it matched the number of oocytes found in wild-type samples (p = 0.7775, T-test; at 22.5 dpc). These results reveal the existence of an unexpected CHK2-independent mechanism that eliminates *Chk2*^*-/-*^ oocytes around birth.

### The lack of CHK2 does not compromise cyst breakdown, but rather delays follicle formation initiation

**Fig 3:**
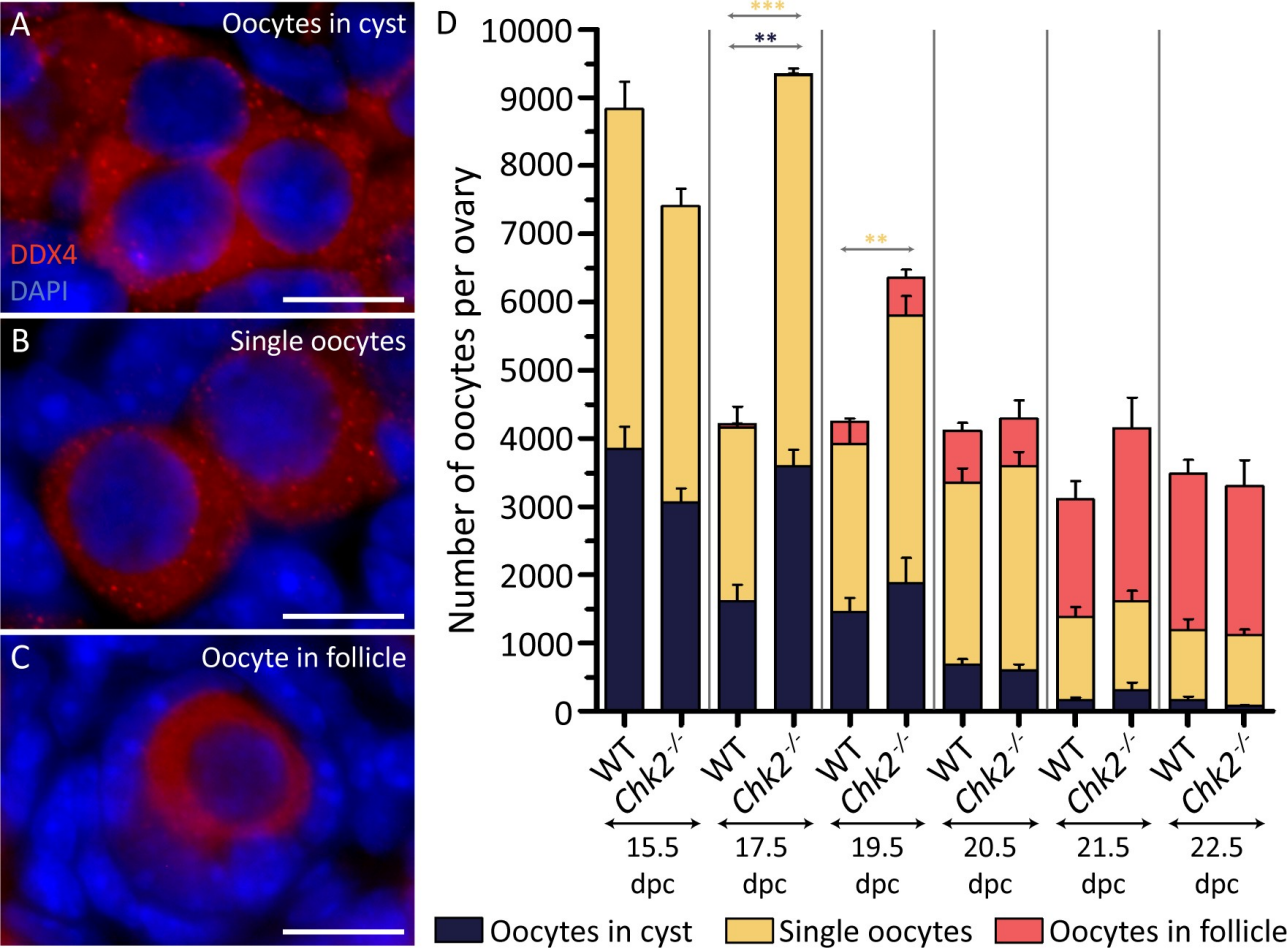
CHK2 seems to be dispensable for cyst breakdown and follicle formation. C) Representative images of oocytes immunostained against the germ-cell marker DDX4 forming a cyst (A), two single oocytes (B) and a follicle (C). The DNA is counterstained with DAPI (blue). The scale bar represents 10 μm. (D) Number of oocytes classified in the three different types described above in wild-type and *Chk2* mutant ovaries of the indicated ages. The lines represent the mean ± SEM. *0.05<p<0.01, **0.01<p<0.001, ***p≤0.001 (Unpaired T-test). The colored significances correspond to the different groups shown in the legend. Data analyzed in this graph come from the same oocytes counted in Fig 2.

Our model also predicts that *Chk2* mutant ovaries will present defects in cyst breakdown and follicle formation, but since *Chk2* mutant mice have been shown to be fertile [30], only minor alterations were expected of these processes. Thus, we classified the oocytes from control and mutant ovaries from 15.5 dpc to 4 dpp into three categories: oocytes in cyst (those sharing cytoplasm with their neighbors, Fig 3A), single oocytes (in which the limit of the oocyte cytoplasm was clearly visible, Fig 3B) and oocytes in follicles (in which the oocytes were surrounded by follicular cells; Fig 3C). At 15.5 dpc, approximately 40% of the oocytes from control and mutant ovaries remained in cysts, while the rest seemed to be individualized (Fig 3D, S3 Table and S1 Data). At 17.5 dpc, fifty percent of the control oocyte population had disappeared (Fig 2C). Remarkably, this translated into the loss of 60% of the oocytes that were forming cysts. However, we also observed the loss of 50% of those oocytes that were already individualized. These data suggest that the massive CHK2-dependent oocyte elimination occurs in both oocytes in cysts and individual oocytes, although other explanations may be plausible (See Discussion). Follicles began to appear in control samples at 17.5 dpc and formed most of the oocyte pool from 3 dpp (21.5 dpc) onwards. In *Chk2* mutants, around 40% of the oocytes seemed individualized at 15.5 dpc. Similar to what we observed in control samples, this proportion persisted to 17.5 dpc. These data suggest that cyst breakdown is not affected by CHK2 ablation. Follicle formation in *Chk2* mutants was very similar to the one found in controls. Follicles first appeared at 17.5 dpc, but in a reduced number and a significantly reduced proportion, as compared to controls (p = 0.0250, T-test; S3 Table). Follicles reached control numbers and proportions in newborn mice (p = 0.7791, T-test; at 19.5 dpc) and constituted most of the oocyte pool from 3 dpp onwards, as in controls. These data suggest that the absence of CHK2 does not principally compromise oocyte cyst breakdown, but rather delays the initiation of follicle formation.

### *Chk2* mutation partially rescues the oocyte loss occurring in *Spo11* mutants and prevents oocyte cyst breakdown

**Fig 4:**
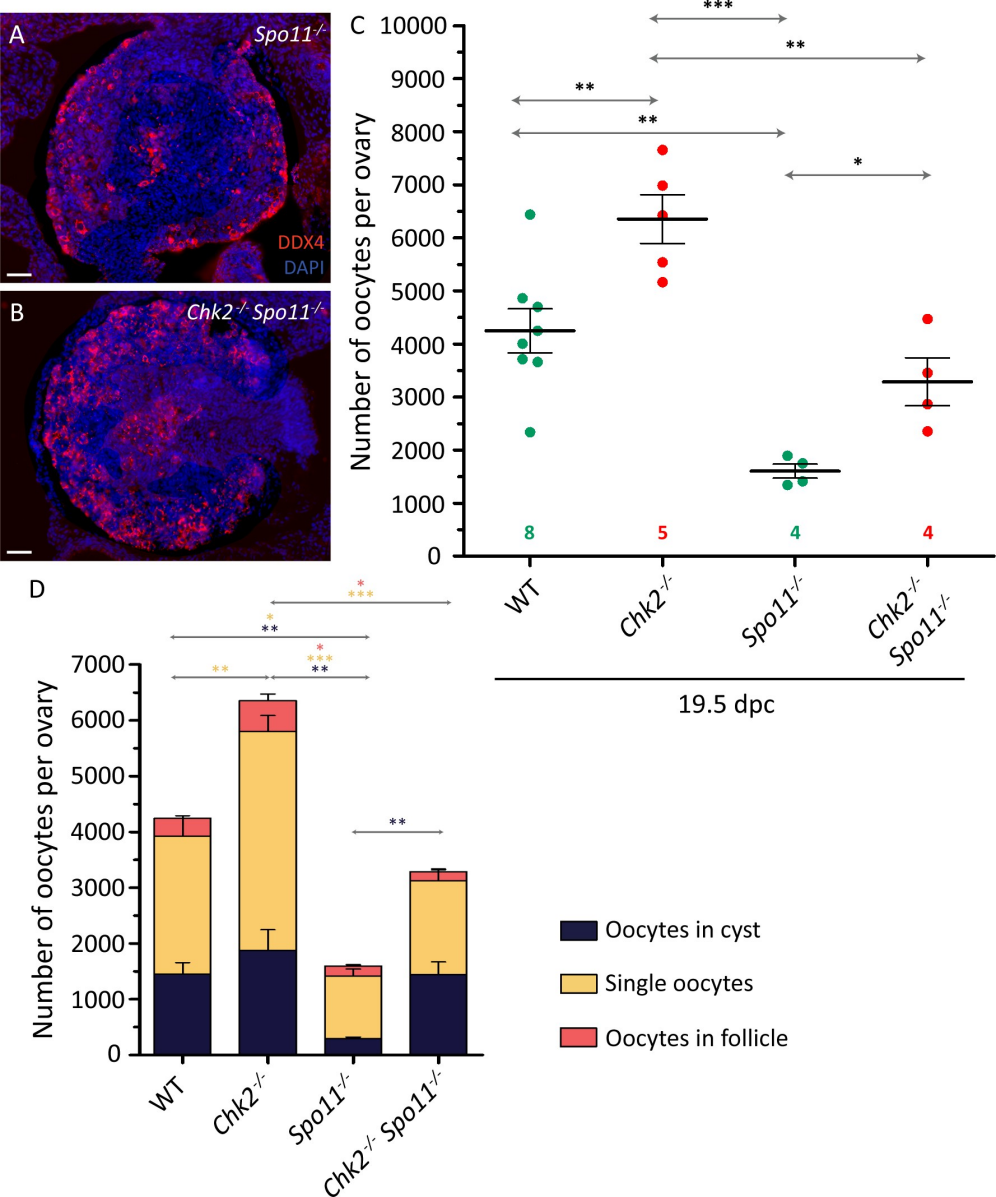
CHK2 rescues the number of oocytes in cyst in a *Spo11* background. (A-B) Representative images of 19.5 dpc *Spo11*^*-/-*^ (A) and *Chk2*^*-/-*^ *Spo11*^*-/-*^ (B) histological sections of ovaries immunostained against DDX4 and counterstained with DAPI. The scale bar represents 40 μm. (C) Number of oocytes in 19.5 dpc control (WT), *Chk2*^*-/-*^, *Spo11*^*-/-*^ and *Chk2*^*-/-*^ *Spo11*^*-/-*^ ovaries. The horizontal lines represent the mean ± the SEM. The data for wild-type and *Chk2*^*-/-*^ samples were taken from Fig 2. *0.05<p<0.01, **0.01<p<0.001, ***p≤0.001 (Unpaired T-test). Data were derived from at least two different animals per condition, and the number of ovaries counted per each genotype and condition is indicated below. (D) Number of oocytes classified in the three different types (cyst, single oocytes, and follicles) from 19.5 dpc control (WT), *Chk2*^*-/-*^, *Spo11*^*-/-*^ and *Chk2*^*-/-*^ *Spo11*^*-/-*^ ovaries. The lines represent the mean ± SEM. The data for wild-type and *Chk2*^*-/-*^ samples were taken from Fig 3. **0.01<p<0.001 (Unpaired T-test). The colored significances correspond to the different groups shown in the legend. Data analyzed in this graph come from the same oocytes counted in C.

At the onset of meiotic prophase, SPO11 generates multiple DSBs that will drive meiotic recombination and homologous chromosome synapsis [13]. Thus, based upon our model, we expected that the massive perinatal oocyte loss occurring in *Spo11* mutants would be independent of CHK2. To test this, the number of oocytes present in newborn ovaries from *Spo11*^*-/-*^ and *Spo11*^*-/-*^ *Chk2*^*-/-*^ mice was counted. *Spo11*^*-/-*^ ovaries presented approximately 25% of the number of oocytes found in *Chk2*^*-/-*^ mice of the same age (p<0.0001, T test, Fig 4, S4 Table). Significantly, *Spo11*^*-/-*^ *Chk2*^*-/-*^ ovaries contained twice as many oocytes as *Spo11*^*-/-*^ ovaries (p = 0.0118, T-test, S4 Table and S1 Data), suggesting that CHK2 was responsible for part of the oocyte death occurring in *Spo11*^*-/-*^ fetal ovaries. *Spo11*^*-/-*^ oocytes have been reported to contain DNA damage at pachynema [31]. Thus, CHK2 may be activated as a response to the presence of this DNA damage, and this may lead to the elimination of some of these oocytes, as previously reported [32]. Importantly, *Spo11*^*-/-*^ *Chk2*^*-/-*^ ovaries contained half the number of oocytes found in *Chk2*^*-/-*^ ovaries (p = 0.0022, T test). These results suggest that although CHK2 participates in *Spo11*^*-/-*^ oocyte elimination, CHK2-independent mechanisms are also responsible for part of the oocyte death occurring in *Spo11*^*-/-*^ fetal ovaries.

Interestingly, the ablation of CHK2 in *Spo11*-mutant mice selectively rescued oocytes in cysts (p = 0.0025, T-test; Fig 4D, S4 Table and S1 Data), suggesting that CHK2 activity is required for the cyst breakdown, at least in the absence of SPO11.

### CHK1 function is required to eliminate oocytes in *Chk2*^*-/-*^ ovaries *in vitro*

**Fig 5:**
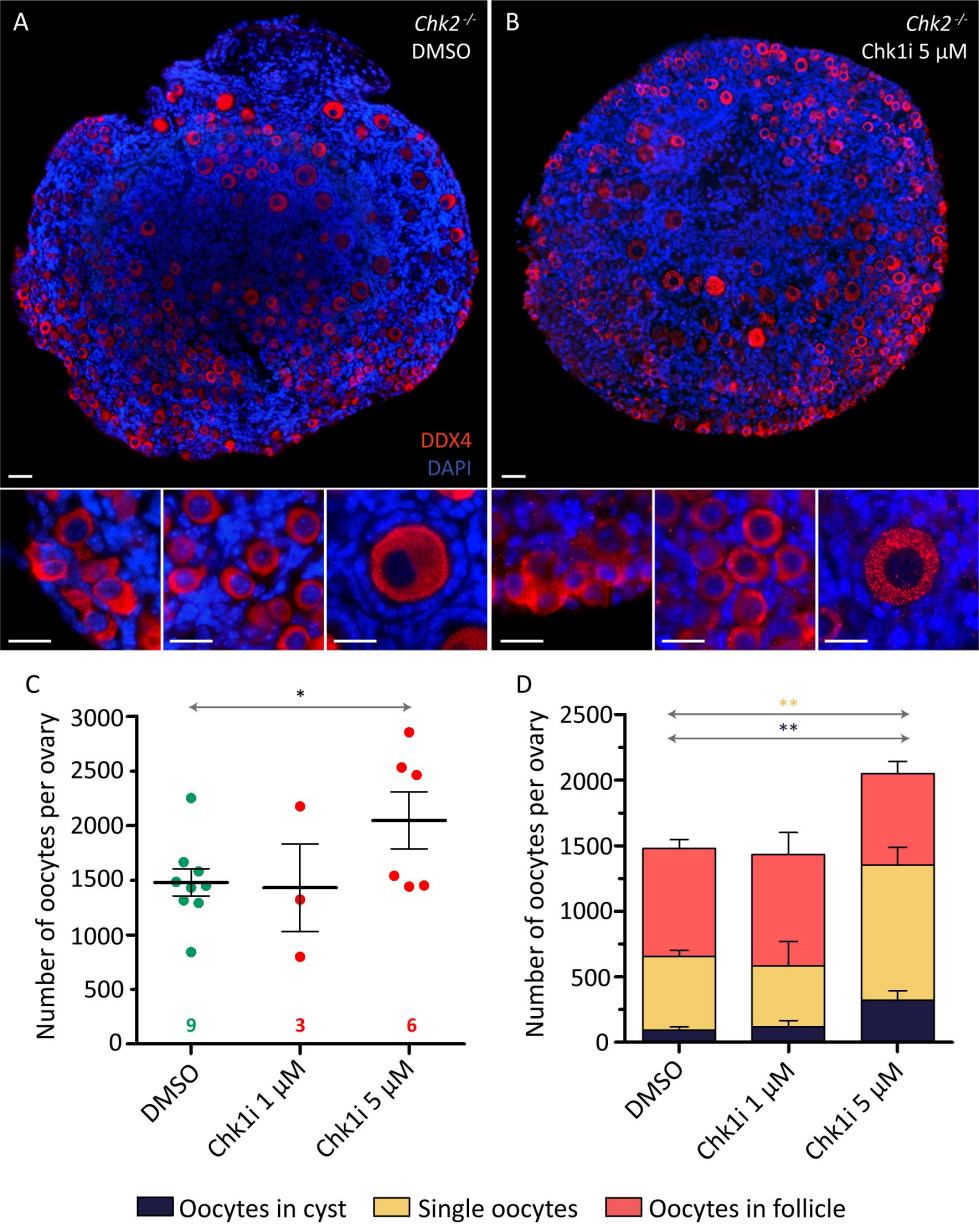
The inhibition of CHK1 *in vitro* rescues the oocyte number in *Chk2*^*-/-*^ ovaries. (A-B) Histological sections of *Chk2*^-/-^ DMSO-treated ovaries (A) and 5 μM LY2603618 (CHK1i)-treated ovaries (B) immunostained against DDX4 and counterstained with DAPI. The inserts show the detail of oocytes in cyst (left), single oocytes (center) and a follicle (right). The scale bar in the top image represents 40 μm and applies to both top images. The scale bar in the insert represents 20 μm and applies to all inserts. (C) Number of oocytes found in *Chk2*^*-/-*^ 19.5 dpc ovaries after five days of culture exposed to DMSO, or 1 μM, or 5 μM of CHK1i. The lines represent the mean ± the SEM. *0.05<p<0.01, **0.01<p<0.001, ***p≤0.001 (Unpaired T-test). Data were derived from at least three different animals per condition, and the number of ovaries counted per each genotype and condition is indicated below. (D) Number of oocytes classified in the three different types (cyst, single oocytes, and follicles) for the same ovaries. The lines represent the mean ± SEM per each category. **0.01<p<0.001, (Unpaired T-test). The colored significances correspond to the different groups shown in the legend. Data analyzed in this graph come from the same oocytes counted in C.

Contrary to what our model predicted, the number of oocytes present in *Chk2*^*-/-*^ ovaries significantly declined after birth, suggesting the existence of an alternative mechanism eliminating oocytes with persistent DNA damage. DDR relies on the activation of two effector kinases, CHK1 and CHK2, to repair the DNA damage, arrest cell-cycle progression, and, if necessary, induce apoptosis [20]. So, we wondered if CHK1 was compensating for the loss of CHK2 and, hence, was responsible for the postnatal elimination of *Chk2*^*-/-*^ oocytes. To test this hypothesis, an organotypic culture was set up that allowed follicle formation *in vitro* (S2 Fig). With this, newborn *Chk2*^*-/-*^ ovarian samples were cultured in the presence of different concentrations of LY2603618, a specific CHK1 inhibitor (CHK1i) [33]. We observed no difference in the number of oocytes found in cultured ovaries under a low concentration of CHK1i (1 μM), as compared to the DMSO controls (p = 0.8815, T-testT test; Fig 5, S5 Table and S1 Data). However, the addition to the culture medium of 5μM LY2603618 resulted in an increased presence of oocytes after culture (p = 0.0473, T-test; Fig 5). These samples contained almost twice as many oocytes as did control ones. Moreover, they contained, on average, three times the proportion of oocytes in cysts, compared to controls (p = 0.0010; T test; Fig 5, S5 Table and S1 Data) and 40% less follicles than controls (p = 0.0001, T test). These differences can be attributed to reduced CHK1 activity since ovaries exposed to 5μM LY2603618 showed a reduced presence of the active form of CHK1 (phosphorylated S296-CHK1) in the cultured oocytes (S3 Fig). Thus, these data suggest that CHK1 eliminates *Chk2*^*-/-*^ oocytes after birth and helps to break down the oocyte cysts to form follicles.

### Fetal oocyte death mediated by LINE-1 activation depends on CHK2

**Fig 6:**
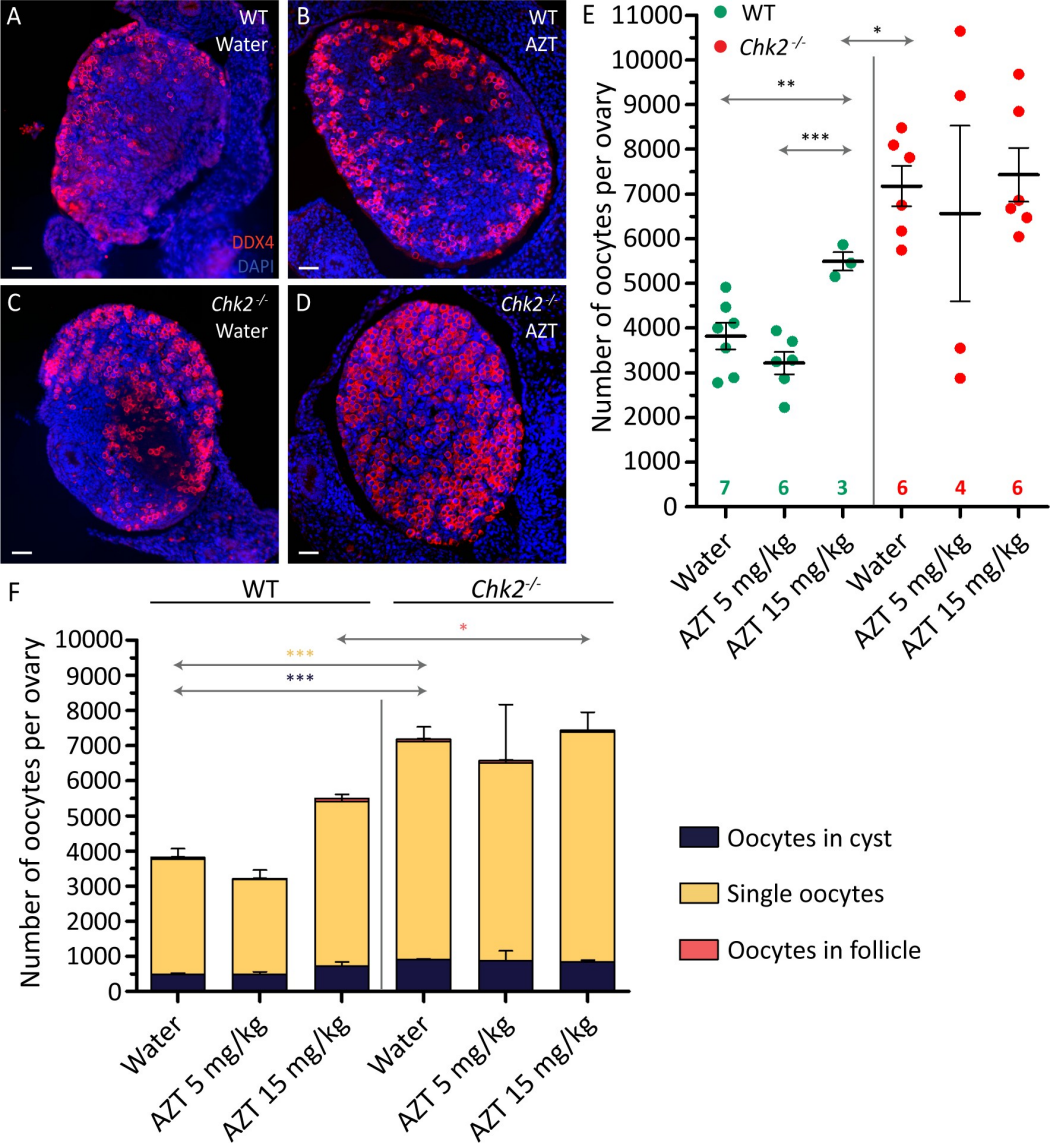
LINE-1 inhibition rescues the oocyte number in wild-type ovaries, but not in *Chk2*^*-/-*^ ovaries. (A-D) Histological sections of control (WT) treated with water (A), AZT-inhibited control (B), *Chk2*^-/-^ treated with water (C) and AZT-inhibited *Chk2*^*-/-*^ (D) ovaries immunostained against DDX4 and counterstained with DAPI. The scale bar represents 40 μm and applies to all images. (E) Number of oocytes per ovary after five days of water or AZT treatment. The round symbols represent the mean, and the lines the SEM. *0.05<p<0.01, **0.01<p<0.001, ***p≤0.001 (Unpaired T-test). Data were derived from at least two different animals per condition, and the number of ovaries counted per each genotype and condition is indicated below. (F) Number of oocytes classified in the three different types (cyst, single oocytes, and follicles) for the same ovaries. The lines represent the mean ± SEM per each category. *0.05<p<0.01, **0.01<p<0.001, ***p≤0.001 (Unpaired T-test). The colored significances correspond to the different groups shown in the legend. Data analyzed in this graph come from the same oocytes counted in E.

The activation of the transposable LINE-1 element triggers fetal oocyte death [11]. Since LINE-1 retrotransposition into the genome may cause DNA damage [34], we wondered if the fetal oocyte death caused by LINE-1 would depend on the activation of DDR. To test this, 11.5 dpc pregnant mice carrying either wild type or *Chk2*^*-/-*^ fetuses were treated for six days with Azidothymidine (AZT, which prevents LINE-1 activation) and fetal ovaries at 17.5 dpc were collected. AZT is a nucleoside analog that specifically inhibits the retrotranscriptase activity required for LINE-1 retrotransposition into the genome. When we tested the previously reported concentration (5mg/Kg, Malki et al., 2014) on wild-type mice, we were unable to observe an effect on the number of oocytes present in the AZT-treated ovaries (p = 0.1577, T-test; Fig 6, S6 Table and S1 Data). Nevertheless, when the dose was raised to 15 mg/kg, the AZT-treated wild-type ovaries contained significantly more oocytes than did water controls (p = 0.0090, T-test; Fig 6E, S6 Table and S1 Data). Interestingly, the treated wild-type ovaries had fewer oocytes than did *Chk2*^*-/-*^ mice treated with water (p = 0.0421, T-test), suggesting that the *Chk2* mutation could rescue more oocytes than could AZT inhibition. When the effect of the AZT treatment on the *Chk2* mutants was analyzed, we did not observe any difference in the number of oocytes recovered in the control or in the AZT-treated samples, at any of the doses used (p = 0.7218 and p = 0.7435, T-test; 5 mM and 15 mM, respectively). These data show that the decrease in the number of oocytes caused by the activation of LINE-1 during fetal development is dependent on CHK2. Also, treatment with AZT had no influence in cyst breakdown or follicle formation in either wild-type or *Chk2* mutant samples (Fig 6F, S6 Table and S1 Data).

## Discussion

**Fig 7:**
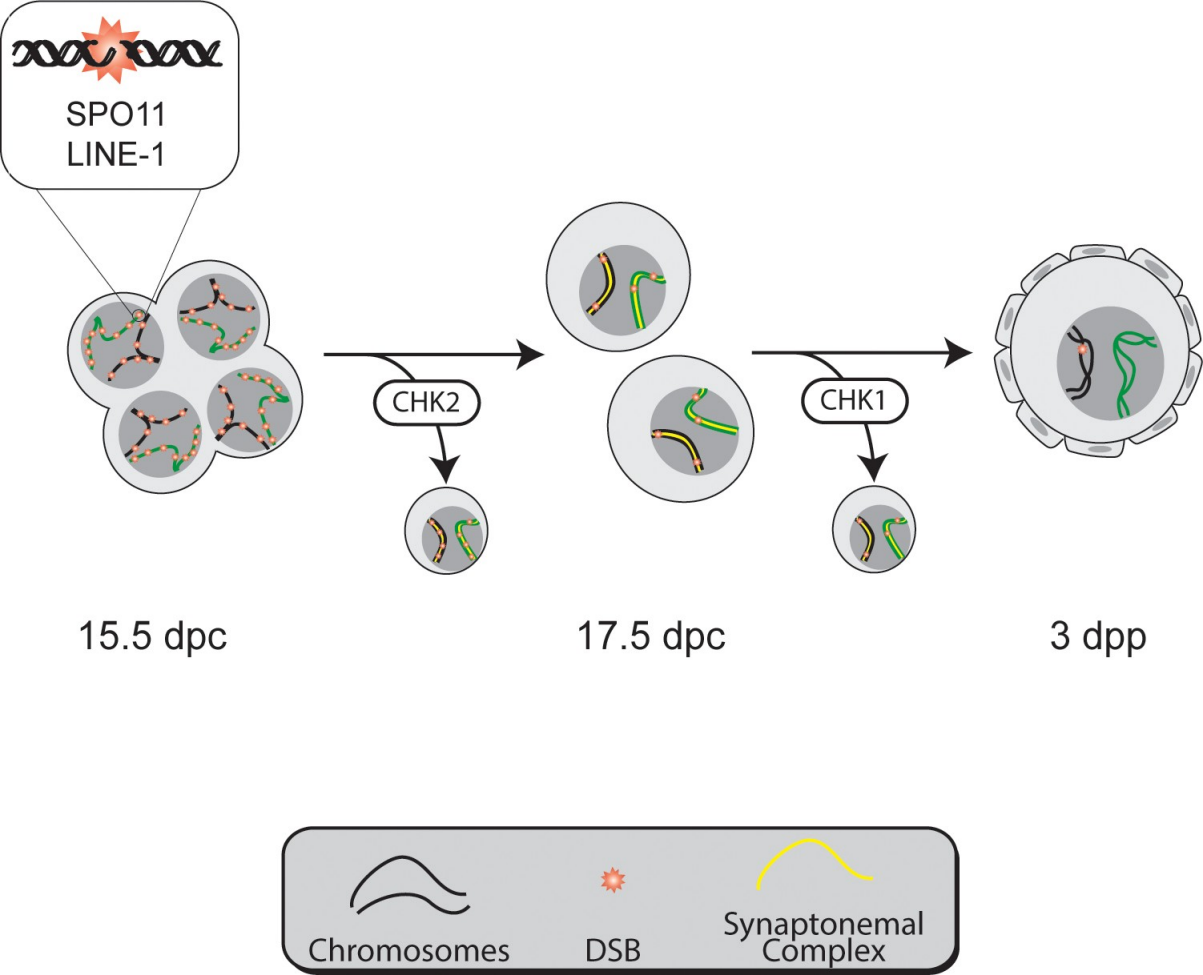
DDR controls the oocyte population around birth. Model showing how DDR regulates the number of oocytes found in mouse ovaries around birth. At 15.5 dpc, oocytes are grouped in cysts and contain extensive DNA damage which can be caused by multiple origins, including SPO11 function and LINE-1 activation. Oocytes with more unrepaired DSBs are eliminated around 16.5 dpc by a CHK2-dependent mechanism. This mechanism helps cyst breakdown and oocyte individualization. After birth, a CHK1-dependent mechanism can eliminate oocytes that accumulate unrepaired DSBs. Ultimately, DDR is required to establish the follicle reserve in mice.

Mammalian female reproduction greatly depends on the ability of the oocytes to arrange themselves into follicles. To do so, oocytes need to disaggregate from the cysts and surround themselves with ovarian stromal cells. In this study, we have uncovered the critical role of DDR in controlling this process in mammals. Our data show that the oocytes that accumulate high levels of unrepaired DSBs at pachynema are eliminated by the CHK2-dependent DDR during fetal development (Fig 7). This mechanism is required to timely initiate folliculogenesis. Moreover, the existence of a CHK1-dependent mechanism eliminating *Chk2* mutant oocytes, which promotes oocyte cyst breakdown and follicle formation, demonstrates the importance of DDR in regulating these early stages of mammalian oogenesis.

Mammalian gametogenesis is sexually dimorphic in several aspects [25]. One of them is the apparently different efficiency in completing meiotic recombination of spermatocytes and oocytes [26,27]. While mammalian spermatocytes complete repair of most of their DSBs at pachynema, oocytes show multiple unrepaired DSBs at this stage [26]. Our findings suggest that the existence of these unrepaired DSBs is crucial to set the oocyte population during fetal development. Thus, DDR is at least partly responsible in determining the perinatal oocyte pool.

What is noteworthy is that our data show that this CHK2-mediated meiotic response to DNA damage can react to SPO11-originated DSBs as well as to other sources of DNA damage. It has been previously reported that *Spo11*^*-/-*^ oocytes present markers of DNA damage [31], and these can activate a CHK2-dependent oocyte elimination [32]. Our results confirm these findings, revealing that DDR is partly responsible for eliminating *Spo11* mutant oocytes. However, the observed rescue in 1 dpp *Spo11*^*-/-*^ *Chk2*^*-/-*^ ovaries does not reach *Chk2*^*-/-*^ numbers, as one would expect if DDR activation was the only mechanism eliminating *Spo11*^*-/-*^ oocytes. In fact, our data show that DDR is not the main mechanism to eliminate *Spo11*^*-/-*^ oocytes during fetal development, since only ~35% of the oocytes are eliminated by a CHK2-dependent pathway. Thus, other mechanisms should account for the elimination of most of the *Spo11* mutant fetal oocyte pool. Presumably, the response to the presence of unsynapsed chromosomes may be responsible for this cell death. At the end of meiotic prophase, unsynapsed chromosomes are silenced by the activation of a cascade of events involving several key proteins of DDR, such as ATR, MDC1 or H2AX, which ultimately leads to their silencing [35–38]. This mechanism, known as meiotic silencing of unsynapsed chromosomes (MSUC), has been shown to be able to eliminate oocytes with asynaptic chromosomes [39]. Thus, it is plausible that most of the *Spo11*^*-/-*^ fetal oocytes are eliminated as a consequence of the MSUC mechanism. This finding demonstrates the existence of different surveillance mechanisms that monitor meiotic prophase progression which are activated by different events, such as asynapsis or the presence of recombination intermediates [15]. Nevertheless, the fact that part of the signaling machinery that participates in the response to synapsis and recombination defects is shared by both pathways (such as, ATR [22,24,40]) makes the study of these mechanisms very complex. Based on the dispensability of CHK2 to eliminate the majority of *Spo11* mutant fetal oocytes, one could think that DDR is not required to activate the mechanism that responds to asynapsis. However, our finding that CHK1 may compensate for the loss of CHK2 urges us to analyze the involvement of CHK1 in the elimination of *Spo11* mutant oocytes.

Recent papers have studied the downstream effectors of DDR in controlling the oocyte population. Canonically, active CHK1 and CHK2 promote the activation of the p53 family members (p53 and p63), which in turn can induce apoptosis by expressing intrinsic apoptosis pathway components (such as *Puma*, *Noxa*, *Bax*). Surprisingly, the ablation of p53 and p63 leads to the complete rescue of the number of oocytes present in recombination-deficient mutants (e.g., *Trip13*^*gt/gt*^) as well as synapsis-deficient mutants (*Spo11*^*-/-*^*)*[41]. Thus, these data suggest that defects in synapsis and recombination activate p53 and p63, which could imply the existence of a common mechanism to control cell-cycle progression in response to these defects. However, a recent publication has shown that the combined ablation of PUMA and NOXA can only rescue the number of oocytes in recombination-deficient mutants, suggesting the existence of genetically separate signaling pathways controlling the oocyte pool in mammals [42]. Altogether, these two recent reports only show the need to investigate the mechanisms that control the mammalian oocyte pool to understand how recombination and synapsis errors in the oocytes are sensed, signaled and responded to.

Apart from synaptic and recombination defects, the activation of the LINE-1 retrotransposon can also trigger fetal oocyte death [12]. During germ-cell development, LINE-1 is derepressed as a consequence of the erasing of the genome methylation marks to initiate the epigenetic reprogramming of the future egg. Restoration of DNA methylation will not take place after birth. Thus, meiotic prophase oocytes suffer from a high expression of LINE-1 [11]. This rise in LINE-1 activation is associated with the loss of approximately 50% of the oocytes, occurring between embryonic days 15.5 and 17.5. To date, it is not clear if this rise in LINE-1 activation results in increased insertions of LINE-1 in the oocyte genome. And, in the same way, it is also not clear what mechanism is associating LINE-1 activation to oocyte death [12]. Since LINE-1 incorporation into the genome may cause DNA damage and the inhibition of LINE-1 activity by AZT was reported to cause an oocyte rescue similar to the one we observed in *Chk2* mutants [11,12], we investigated whether CHK2 was required for the oocyte loss caused by LINE-1 activity. We found problems recapitulating the previously published results [11], and had to triplicate the dose of AZT administered to our wild-type mice to observe a rescue of oocytes. Recently, a correction of the original paper was made stating the actual dose used for the AZT treatment (50mg/ml) [43], which was more than twice the one we used. Importantly, this explains why the rescue was not complete, as was previously reported. Nonetheless, it showed that LINE-1 activation was responsible for at least part of the fetal oocyte death that occurs in wild-type mice. However, AZT treatment had no effect on *Chk2*-mutant mice, suggesting that the oocyte death originated by LINE-1 was also dependent on CHK2. We do not think this is related to the low AZT concentration used because a recent report has shown that exposition to 50 mg/ml of AZT to *Chk2*^*-/-*^ fetal oocytes has no effect on the number of oocytes found at 17.5 dpc [44]. Based upon these data, we propose that the mechanism behind LINE-1-induced fetal oocyte death, at least up to 17.5 dpc, is the formation of DNA damage, which ultimately leads to the activation of CHK2 and the elimination of fetal oocytes.

Our data also reveal the unexpected involvement of CHK1 in the elimination of oocytes with persistent DNA damage. The study of the function of CHK1 during meiotic prophase has been challenging because CHK1 is required to allow embryo development in mammals [45]. Furthermore, the use of conditional mutants has shown that CHK1 is also required for germ-cell proliferation [23]. Our *in vitro* approach allowed us to show that CHK1 activity, in either the oocytes and/or the somatic cells of the ovary, is able to compensate for the loss of CHK2. This finding explains why the number of oocytes found in 4 dpp *Chk2*^*-/-*^ ovaries is the same as those in wild-type ovaries. The fact that *Chk2* mutants have twice the number of oocytes as do controls at 17.5 dpc suggests that CHK1 activation is delayed, as compared to CHK2. Consistent with this idea, we found that *Chk2*^*-/-*^ early-pachytene oocytes had more unrepaired DSBs than did wild-type oocytes, suggesting that CHK1 may not be active at early-pachynema. Nonetheless, the differences between the two genotypes disappeared as cells completed meiotic prophase, so that late-diplotene wild-type and *Chk2* mutant oocytes presented the same number of unrepaired DSBs. Interestingly, we did not see a significant change in the oocyte population of control mice from 19.5 dpc to 22.5 dpc. To us, this suggests that the CHK1-dependent perinatal loss of oocytes is negligible in CHK2-proficient mice. Agreeing with this hypothesis, a recent study reports that CHK1 is not activated in perinatal ovaries from wild-type mice [41].

These findings open the possibility that CHK2 and CHK1 may be differentially regulated during meiotic prophase. In fact, sensitivity to DNA damage differs significantly from early-prophase to dictyate-arrested oocytes. While leptotene oocytes endure hundreds of DSBs, dictyate oocytes are very sensitive to DNA damage [27,46]. This is partly accomplished by regulating the activity of TAp63α, a key protein of DDR in oocytes [47]. p63 is required to eliminate dictyate-arrested oocytes weakly irradiated (0.3 Gy [46]). However, p63 is not expressed until oocytes reach mid-meiotic prophase (17 dpc onwards) and its function is actively inhibited [47], suggesting that p63 activity might be deleterious for meiotic prophase oocytes. Thus, it is plausible that different pathways of DDR are selectively regulated during oogenesis in order to first allow meiotic DSB formation and repair to promote homolog synapsis and crossover formation. Once this is accomplished, DDR may activate alternative, or supplementary, pathways to achieve a greater sensitivity to DNA lesions, in order to assure only high-quality oocytes can pass their genetic information on to the next generation. Thus, we propose CHK2 may participate in the surveillance mechanisms that control the progression of DSB repair until mid-pachynema. This mechanism is responsible for the elimination of approximately 50% of the oocytes during fetal oogenesis. Once this meiotic surveillance mechanism is met, an alternative or supplementary CHK1-dependent pathway may become active to ensure the genetic integrity of the remaining oocytes. This model would explain why CHK1 cannot compensate for the loss of CHK2 during fetal development but can do it later on, once the oocytes may have reached a particular stage (late-pachynema, diplonema and/or dictyate).

The perinatal massive oocyte death that naturally occurs in mouse oogenesis has been historically associated with cyst breakdown [9,10,48]. Germ cells, before differentiating into oocytes, go through several mitotic divisions in which cytokinesis is not completed, thus resulting in a syncitium of cells, the cysts, which will initiate meiosis. At the end of the meiotic prophase, these cysts need to be disassembled so single oocytes can be surrounded by stromal ovarian cells to form follicles. The significance of proper cyst breakdown is manifested in mutants that fail to individualize oocytes, which are infertile due to the formation of follicles containing multiple oocytes [49]. Our model predicted that, at least in part, fetal oocyte loss would facilitate cyst breakdown. So, we expected that, in mutants that fail to activate DDR, cyst breakdown and follicle formation would be altered.

Our data suggest that in our colony less than 45% of the oocytes of 15.5 dpc mice are forming cysts. However, this seems unlikely since cyst breakdown should not start until 17.5 dpc [10]. Nonetheless, some reports show the existence of cyst fragmentation during fetal development that may result in the individualization of oocytes before 17.5 dpc [50]. Notwithstanding, this event seems to be rare during fetal development. Previous studies have shown that less than 7% of the oocytes were individualized at 17.5 dpc, and this figure only reaches 20% by birth [50]. Thus, it is more likely that our data reflect that the approach we used for this analysis is unable to detect all oocytes in cysts. This is not unlikely, especially because of the two-dimensional analysis that we have performed, we were only able to determine whether one oocyte was in a cyst if its sister cells were on the same focal plane. Thus, all oocytes in a cyst in which their sister cells were located in different focal planes may have been scored as individual oocytes. This will ultimately cause an overrepresentation of this category in our dataset.

Taking this caveat into consideration, to address if DDR is required to breakdown cysts and if this had an effect on follicle formation, firstly we will focus our analysis in follicle formation. The analysis of the *Chk2*^*-/-*^ ovaries shows that CHK2 is required to timely initiate follicle formation at 17.5 dpc, but it seems dispensable for cyst breakdown and follicle formation from that time onwards, since the number and proportion of follicles formed in *Chk2* mutants are indistinguishable to those found in control samples. Interestingly, inhibition of the CHK1 function in *Chk2* mutant ovaries reduced the proportion of follicles formed *in vitro* (S5 Table and S1 Data). Furthermore, these samples contained more oocytes in cysts, suggesting that DDR was required for cyst breakdown. In our opinion, these data point out the importance of a functional DDR for the proper timing of follicle formation. Moreover, it suggests that DDR-dependent oocyte elimination may participate in cyst breakdown and follicle formation. In this sense, in *Spo11*^*-/-*^ ovaries, *Chk2* mutation preferentially rescues the number of oocytes in cysts, supporting the idea that DDR impacts cyst breakdown, and consequently follicle formation.

Altogether, our data highlight the importance of DDR in regulating the follicle reserve in the females. First, during fetal development regulating the number of fetal oocytes using a CHK2-dependent response, and postnatally activating a CHK1-dependent response. The oocyte loss caused by these two mechanisms promotes cyst breakdown, facilitating follicle formation and, thus, regulating the reserve of oocytes that mammalian females will use during their entire reproductive lifespan.

## Material and methods

### Animals and genotyping

*Chk2* and *Spo11* mutant mice were generated previously [13,30]. These alleles were maintained in a C57BL6-129Sv mixed background. All experiments were performed using at least two mutant animals and compared with control littermates or with animals of closely related parents. The term wild-type (WT) in the text and figures refers to both homozygous and heterozygous mice. All animals were sacrificed using CO_2_ or decapitation methods following the CEEAAH 1091 protocol (DAAM6395) approved by the Ethics Committee of the Universitat Autònoma de Barcelona and the Catalan Government.

Mouse genotyping was performed by PCR analysis from the DNA extracted from the tails as previously performed [17,18,22].

### Harvesting of the ovaries

Ovarian samples were obtained from females at different ages from 15.5 dpc to 22.5 dpc. The presence of a vaginal plug was used to determine the age of the mice. Females were caged overnight with males and if the vaginal plug was found next morning the female was isolated and defined as 0.5 dpc. To obtain 15.5 dpc and 17.5 dpc ovaries, pregnant females were sacrificed and the fetuses were removed, rinsed in PBS and ovaries were harvested under a stereo microscope (Nikon SMZ-1). Since, in our mouse colony, deliveries always occurred at 19 dpc, neonatal ovaries from 1 dpp to 4 dpp correspond to 19.5 dpc to 22.5 dpc. In other publications, 19.5dpc can also be considered as P0 and 22.5dpc as P3.

### Histology, immunolabeling and oocyte quantification

Harvested perinatal ovaries (from 15.5 dpc to 22.5 dpc) were immediately fixed overnight in 4% paraformaldehyde in PBS. Samples were then dehydrated, cleared and embedded in paraffin using standard procedures. The whole ovary was sectioned at a thickness of 7 μm and a half ovary (every other section) was processed for immunostaining as follows: the sections were deparaffinized and antigen was unmasked by treating the slides for 20 minutes in Sodium Citrate buffer (10 mM Sodium Citrate, 0.05% Tween 20 in Milli-Q water, pH 6.0) at 95°C-100°C. Standard immunofluorescence was then performed using rabbit anti-DDX4 (Abcam) at 1:100 dilution or rabbit anti-Phospho-Chk1 (Ser296) (ThermoFisher) at 1:100 dillution and a mouse monoclonal antibody against p63 (Abcam) at 1:100 dillution. Slides were counterstained with DAPI.

Oocytes were manually counted and classified under the fluorescence microscope (Zeiss Axipohot) with the 63x magnification. Classification of the analyzed oocytes was done as follows: oocytes in cyst (sharing the cytoplasm, Fig 3A), single oocytes (individual oocytes showing clear cytoplasmic limits, Fig 3B) and oocytes in follicles (oocytes surrounded by one layer of flat granulosa cells, Fig 3C). Only DDX4-positive oocytes with a visible nucleus stained with DAPI were considered for the analysis.

### Surface oocyte spreads and immunolabeling

Isolated ovaries from 17.5 and 19.5 dpc mice were placed in a 24-well plate with M2 medium (Sigma-Aldrich) and digested in collagenase dissolved in M2 medium for 20 minutes at 37°C. Then, the ovaries were transferred to hypotonic buffer (30 mM Tris-HCl pH = 8.2, 50 mM Sucrose, 17 mM Sodium Citrate, 5 mM EDTA, 0.5 mM DTT [Roche Diagnostics], 1x Protease Inhibitor Cocktail [Roche Diagnostics] in Milli-Q water) and incubated for 30 minutes at room temperature. The oocytes were released from the ovary by pipetting up and down in 100 mM sucrose and transferred onto slides with fixative (1% PFA, 5 mM Sodium Borate, 0.15% Triton X-100, 3 mM DTT, 1x Protease Inhibitor Cocktail in Milli-Q water, pH = 9.2) in a humid chamber. After two hours of fixation, slides were air-dried, washed with 0.4% Photoflo (Kodak), air-dried again and stored at -80°C until use.

Immunostaining was performed using standard methods [26] with the following primary antibodies: rabbit anti-SYCP3 (Abcam) at 1:200 dilution and mouse anti-γH2AX (Millipore) 1:200 dilution.

### Neonatal ovarian organ culture

Neonatal mouse ovaries were harvested and cultured, as previously reported [51]. Briefly, ovaries were placed in prewarmed dissection media (Leibowitz L15 [Sigma-Aldrich] with 3mg/mL of BSA) and, under the stereomicroscope, they were trimmed with forceps, eliminating the remains of the bursal sac, the oviduct, and the uterus. Ovaries were transferred to UV- sterilized polycarbonate membrane (Whatman) sitting on a 24-well plate containing prewarmed culture media (α-Minimum Essential Medium (Thermofisher) with 3mg/mL of BSA). The samples were treated with 1 μM or 5 μM of the CHK1 inhibitor LY2603618 (Selleckchem), dissolved in DMSO. Control samples were treated with equivalent volumes of DMSO. All ovaries were cultured in an incubator at 37°C supplied with 5% CO_2_ for five days. After this time, ovaries were processed as previously mentioned to obtain ovarian sections.

### AZT treatment

To inhibit LINE-1 activity, 5mg/kg/day and 15 mg/kg/day AZT (Sigma Aldrich) were administered to wild-type and *Chk2*^*-/-*^ pregnant mice from 11.5 dpc until 17.5 dpc by gavage, as described elsewhere [11]. At 17.5 dpc, pregnant mice were sacrificed and fetal ovaries were harvested and processed as mentioned above to obtain ovarian sections.

### Image processing and data analysis

Microscopy analysis was performed with a Zeiss Axiophot microscope. Images were captured with a Point Gray Research, Inc. camera with the ACO XY Software (A. COLOMA Open microscopy).

All images were processed with Adobe Photoshop CC to overlay the different fluorescent channels and match the intensity observed in the microscope. The software Image Composite Editor (https://www.microsoft.com/en-us/research/product/computational-photography-applications/image-composite-editor/) was used to stitch the composite panoramic images of the sections.

### Statistical analysis

Data analysis and statistical inference were performed using the GraphPad Prism 5 software (https://www.graphpad.com/scientific-software/prism/).

## Supporting information

S1 Fig Meiotic prophase progression is faster in the absence of CHK2. Representative oocytes at leptotene, zygotene, pachytene and diplotene stained against the axial element protein SYCP3 (green). The scale bars represents 10 μm. Percentage of oocytes found in each meiotic prophase stage in control (WT, N = 2) and *Chk2* mutant ovaries (N = 2) from 17.5 and 19.5 dpc mice is shown.
(TIF)

S2 Fig The organotypic culture allows folliculogenesis initiation *in vitro*. (A) Number of oocytes classified in the three different types (cyst, single oocytes, and follicles) for control ovaries cultured for different number of days. After five days of culture, the ovaries present the same percentage of follicles as the 21.5 dpc ovaries (3 dpp). (B) Histological section of a control ovary after five days of culture, immunostained against DDX4 and counterstained with DAPI. The scale bar represents 40 μm.
(JPG)

S3 Fig Exposure to 5μM LY2603618 causes a reduction of the active form of CHK1 in cultured oocytes. (A-D) Representative images of *Chk2* mutant ovaries cultured in the presence of DMSO (A-B) or 5μM LY2603618 (C-D) stained against the oocyte marker, p63, and the phosphorylated Ser296-CHK1 (pCHK1). Panels B and D show the pCHK1 signal from images displayed in Panels A and C, respectively. Notice how the intensity of the signal from DMSO-treated samples is significantly stronger than the one present in CHKi-treated samples. Also, a few discrete foci can be observed in the nucleus of DMSO-treated oocytes that appear to be absent from CHK1i-treated oocytes. (E) Quantification of the intensity of the pCHK1 signal present in the nucleus of DMSO- and CHK1i-treated oocytes. Cells were obtained from three mice per each condition. N denotes the number of oocytes counted and the p value is the significance of the T-test analysis.
(TIF)

S1 Data The file containing the detailed statistics for all comparisons performed in this manuscript. (XLSX)

S1 Table Number of γH2AX patches per oocyte at pachynema and diplonema from wild-type and *Chk2*^*-/-*^ ovaries. (DOCX)

S2 Table Oocyte number on the different perinatal days from wild-type and *Chk2*^-/-^ ovaries. (DOCX)

S3 Table Number and percentage of the different classes of oocytes found on the wild-type and *Chk2*^-/-^ ovaries analyzed. (DOCX)

S4 Table Number and percentage of the different classes of oocytes found at 19.5 dpc ovaries from the different indicated genotypes. (DOCX)

S5 Table Number and percentage of the different classes of *Chk2*^*-/-*^ oocytes found after culture at the indicated conditions. (DOCX)

S6 Table Number and percentage of the different classes of oocytes found at 17.5 dpc after AZT treatment. (DOCX)
