# Supplementary figures and images for: The DNA damage response is required for oocyte cyst breakdown and follicle formation in mice
Source: PLoS Genet. 2020 Nov 18;16(11):e1009067. doi: 10.1371/journal.pgen.1009067 (PMC7710113; doi:10.1371/journal.pgen.1009067)

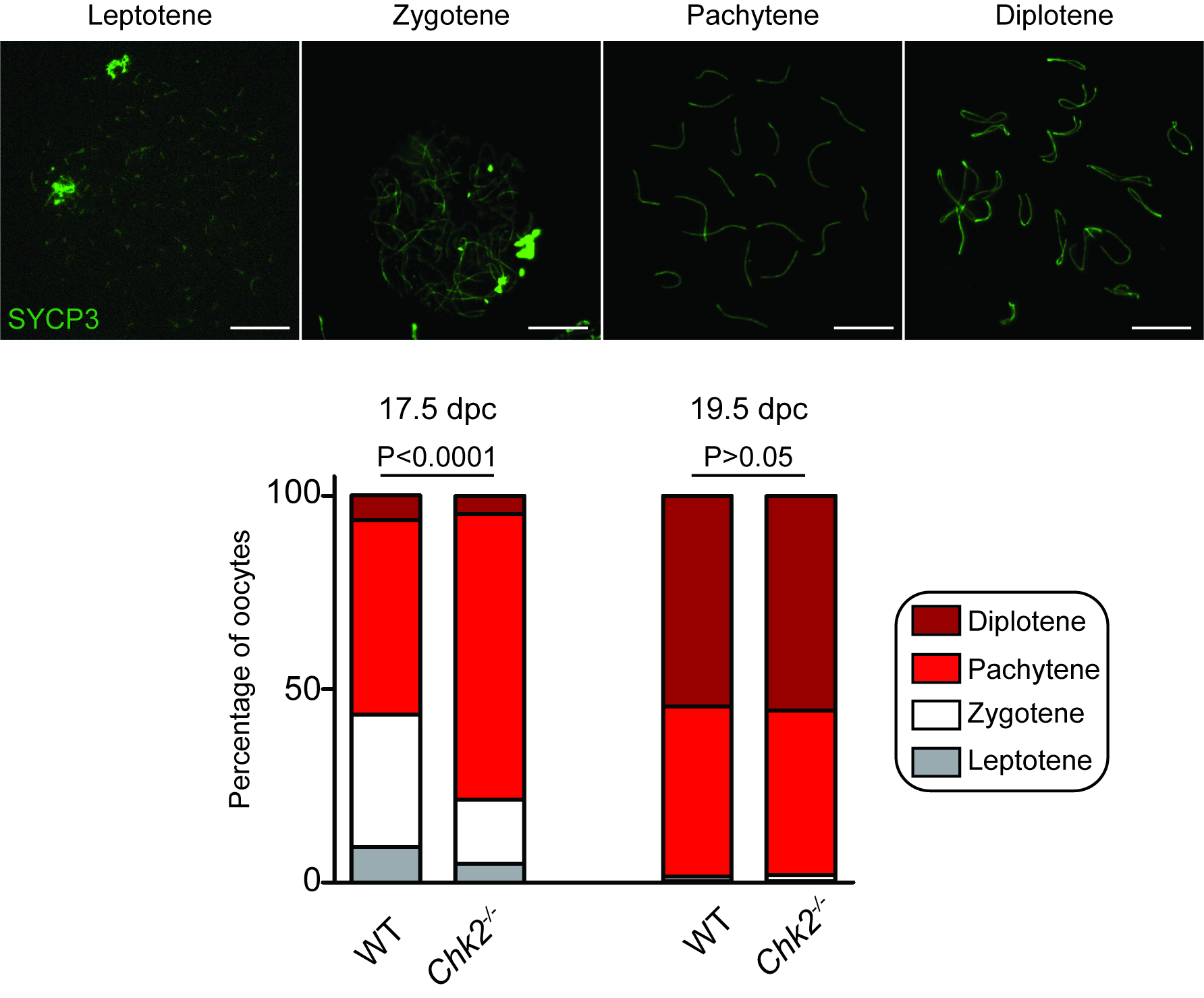

Supplement: S1 Fig — Representative oocytes at leptotene, zygotene, pachytene and diplotene stained against the axial element protein SYCP3 (green). The scale bars represents 10 μm. Percentage of oocytes found in each meiotic prophase stage in control (WT, N = 2) and Chk2 mutant ovaries (N = 2) from 17.5 and 19.5 dpc mice is shown. (TIF) [file pgen.1009067.s001.tif]

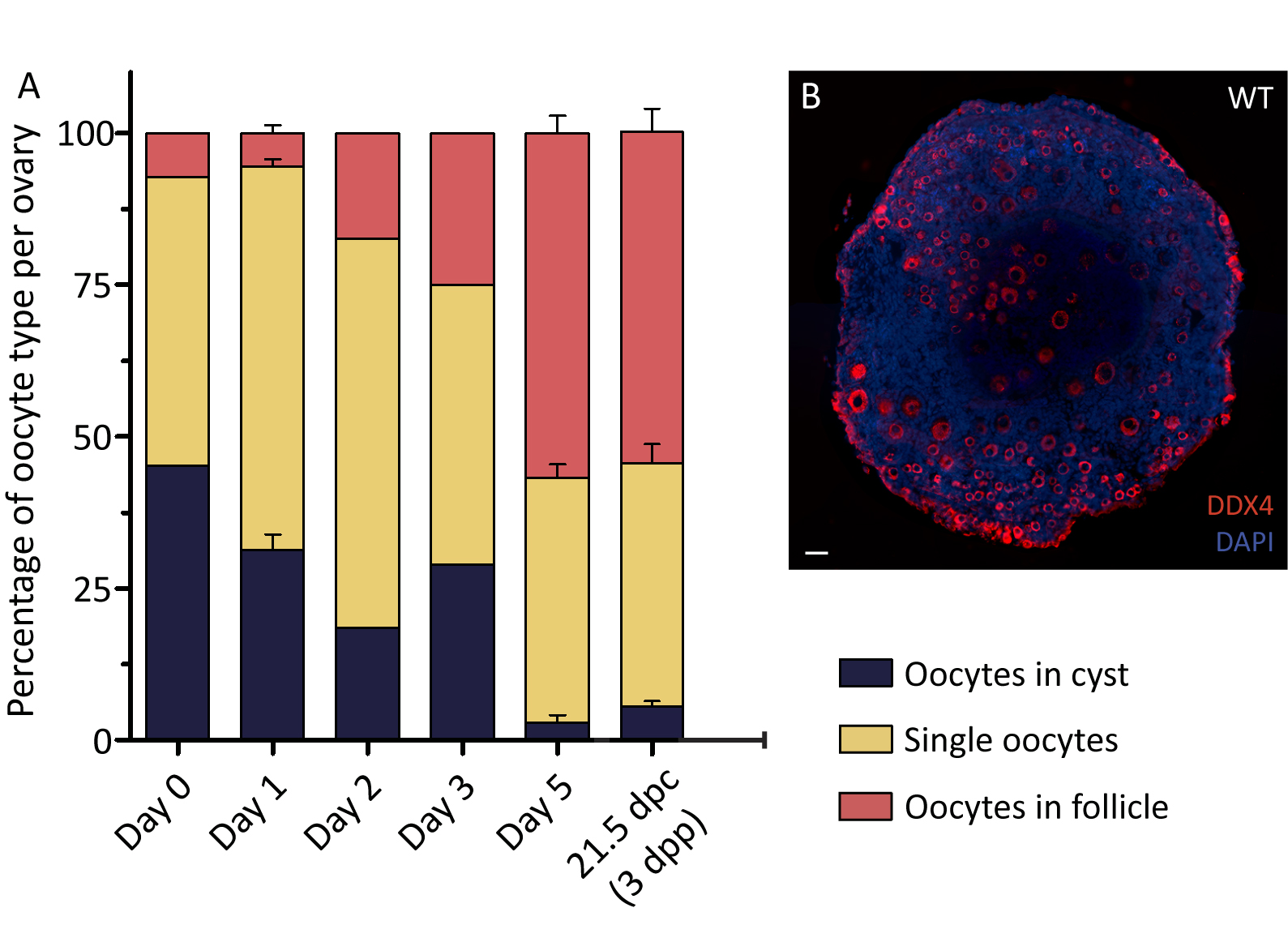

Supplement: S2 Fig — (A) Number of oocytes classified in the three different types (cyst, single oocytes, and follicles) for control ovaries cultured for different number of days. After five days of culture, the ovaries present the same percentage of follicles as the 21.5 dpc ovaries (3 dpp). (B) Histological section of a control ovary after five days of culture, immunostained against DDX4 and counterstained with DAPI. The scale bar represents 40 μm. (JPG) [file pgen.1009067.s002.jpg]

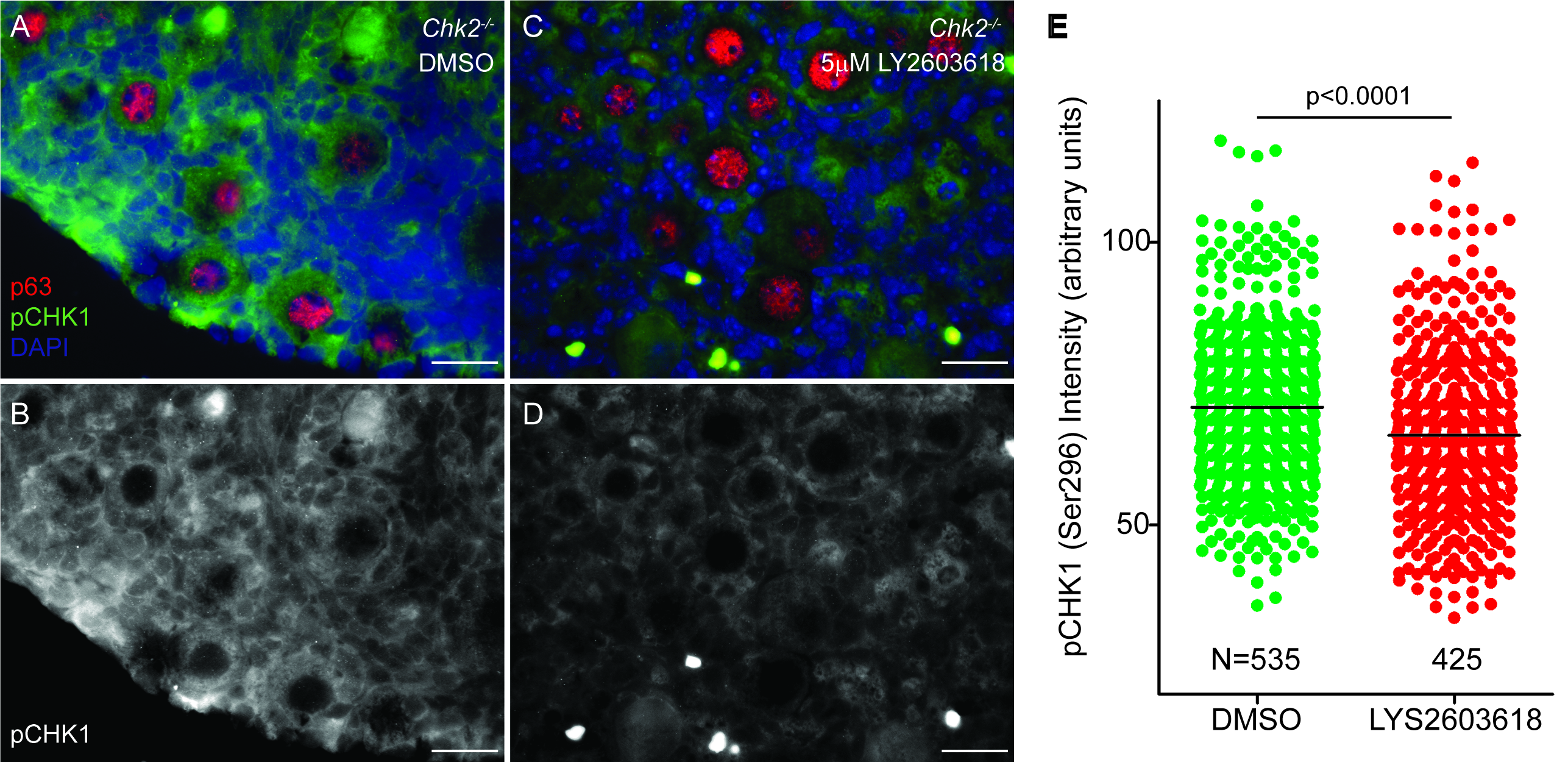

Supplement: S3 Fig — (A-D) Representative images of Chk2 mutant ovaries cultured in the presence of DMSO (A-B) or 5μM LY2603618 (C-D) stained against the oocyte marker, p63, and the phosphorylated Ser296-CHK1 (pCHK1). Panels B and D show the pCHK1 signal from images displayed in Panels A and C, respectively. Notice how the intensity of the signal from DMSO-treated samples is significantly stronger than the one present in CHKi-treated samples. Also, a few discrete foci can be observed in the nucleus of DMSO-treated oocytes that appear to be absent from CHK1i-treated oocytes. (E) Quantification of the intensity of the pCHK1 signal present in the nucleus of DMSO- and CHK1i-treated oocytes. Cells were obtained from three mice per each condition. N denotes the number of oocytes counted and the p value is the significance of the T-test analysis. (TIF) [file pgen.1009067.s003.tif]
