# Supplementary material for: The DNA damage response is required for oocyte cyst breakdown and follicle formation in mice
Source: PLoS Genet. 2020 Nov 18;16(11):e1009067. doi: 10.1371/journal.pgen.1009067 (PMC7710113; doi:10.1371/journal.pgen.1009067)
Supplement: S1 Table — (DOCX) [file pgen.1009067.s005.docx]

| **Stage** | **WT** | ***Chk2 ^-/-^*** |
| --- | --- | --- |
| **Early-pachynema** | 52.9 ± 1.8* (N=50) | 59.3 ± 1.6* (N=50) |
| **Late-pachynema** | 25.3 ± 1.1* (N=75) | 33.3 ± 1.1* (N=75) |
| **Early-diplonema** | 14.9 ± 0.8* (N=36) | 17.5 ± 0.9* (N=36) |
| **Late-diplonema** | 4.7 ± 0.6 (N=39) | 4.4 ± 0.5 (N=39) |
| The numbers express the average ± SEM.  N indicates the number of oocytes counted.  *represents the statistical difference between the two genotypes (T-test). | | |
