# Supplementary material for: The DNA damage response is required for oocyte cyst breakdown and follicle formation in mice
Source: PLoS Genet. 2020 Nov 18;16(11):e1009067. doi: 10.1371/journal.pgen.1009067 (PMC7710113; doi:10.1371/journal.pgen.1009067)
Supplement: S2 Table — (DOCX) [file pgen.1009067.s006.docx]

| **Day** | **WT** | ***Chk2 ^-/-^*** |
| --- | --- | --- |
| **15.5 dpc** | 8832 ± 652.4 (N=5) | 7407 ± 345.9 (N=4) |
| **17.5 dpc** | 4207 ± 556.6* (N=4) | 9344 ± 228.3* (N=4) |
| **19.5 dpc (1 dpp)** | 4246 ± 416.8* (N=8) | 6354 ± 457.2* (N=5) |
| **20.5 dpc (2 dpp)** | 4117 ± 169.1 (N=6) | 4290 ± 382.9 (N=4) |
| **21.5 dpc (3 dpp)** | 3108 ± 405.4 (N=6) | 4145 ± 710.7 (N=4) |
| **22.5 dpc (4 dpp)** | 3480 ± 384.9 (N=8) | 3297 ± 440.1 (N=4) |
| The numbers express the average ± SEM.  N indicates the number of oocytes counted.  *represents the statistical difference between the two genotypes (T-test). | | |
