# Supplementary material for: The DNA damage response is required for oocyte cyst breakdown and follicle formation in mice
Source: PLoS Genet. 2020 Nov 18;16(11):e1009067. doi: 10.1371/journal.pgen.1009067 (PMC7710113; doi:10.1371/journal.pgen.1009067)
Supplement: S3 Table — (DOCX) [file pgen.1009067.s007.docx]

| **Day** | **Genotype** |  | **Oocytes in cyst** | **Single oocytes** | **Oocytes in follicles** |
| --- | --- | --- | --- | --- | --- |
| **15.5 dpc** | **WT (N=5)** | # | 3844 ± 335.3 | 4987 ± 398.8 | 0 |
|  |  | % | 43.6 ± 2 | 56.4 ± 2 | 0 |
|  | ***Chk2*^-/-^ (N=4)** | # | 3057 ± 210.5 | 4351 ± 251.7 | 0 |
|  |  | % | 41.2 ± 2 | 58.8 ± 2 | 0 |
| **17.5 dpc** | **WT (N=4)** | # | 1620 ± 236.2* | 2539 ± 310.9* | 48.6 ± 18 |
|  |  | % | 38.2 ± 1.4 | 60.7 ± 1.4 | 1.1 ± 0.3* |
|  | ***Chk2*^-/-^ (N=4)** | # | 3591 ± 242.9* | 5735 ± 105.8* | 19.3 ± 7.5 |
|  |  | % | 38.3 ± 1.8 | 61.5 ± 1.8 | 0.2 ± 0.1* |
| **19.5 dpc**  **(1 dpp)** | **WT (N=8)** | # | 1456 ± 203.6 | 2466 ± 323.9* | 324.3 ± 46.8 |
|  |  | % | 34.3 ± 4.1 | 57.9 ± 3.7 | 7.8 ± 1.1 |
|  | ***Chk2*^-/-^ (N=5)** | # | 1875 ± 372.7 | 3929 ± 283.2* | 550.8 ± 119.8 |
|  |  | % | 28.9 ± 4.6 | 62.7 ± 5.5 | 8.3 ± 1.3 |
| **20.5 dpc (2 dpp)** | **WT (N=6)** | # | 677.7 ± 86.2 | 2675 ± 207.1 | 763.8 ± 117.5 |
|  |  | % | 16.5 ± 2 | 64.9 ± 3.9 | 18.6 ± 3 |
|  | ***Chk2*^-/-^ (N=4)** | # | 593.8 ± 90.9 | 3005 ± 202.3 | 691.4 ± 269.7 |
|  |  | % | 14.6 ± 3.3 | 70.5 ± 2.7 | 14.9 ± 5 |
| **21.5 dpc (3 dpp)** | **WT (N=6)** | # | 167.7 ± 32.07 | 1212 ± 149.4 | 1728 ± 271.2 |
|  |  | % | 5.6 ± 0.9 | 40 ± 3.1 | 54.5 ± 3.7 |
|  | ***Chk2*^-/-^ (N=4)** | # | 304.1 ± 118.4 | 1308 ± 153 | 2533 ± 458.1 |
|  |  | % | 6.4 ± 1.8 | 32.9 ± 3.5 | 60.7 ± 2.6 |
| **22.5 dpc (4 dpp)** | **WT (N=8)** | # | 163.9 ± 47.9 | 1028 ± 158.2 | 2288 ± 205.2 |
|  |  | % | 4.2 ± 0.8 | 29 ± 1.8 | 66.8 ± 2.4 |
|  | ***Chk2*^-/-^ (N=4)** | # | 74 ± 14.07 | 1040 ± 84.3 | 2183 ± 381.7 |
|  |  | % | 2.3 ± 0.4 | 32.6 ± 3.3 | 65.1 ± 3.6 |
| The numbers express the average ± SEM.  N indicates the number of oocytes counted.  *represents the statistical difference between the two genotypes (T-test). | | | | | |
