# Supplementary material for: The DNA damage response is required for oocyte cyst breakdown and follicle formation in mice
Source: PLoS Genet. 2020 Nov 18;16(11):e1009067. doi: 10.1371/journal.pgen.1009067 (PMC7710113; doi:10.1371/journal.pgen.1009067)
Supplement: S4 Table — (DOCX) [file pgen.1009067.s008.docx]

| **Genotype** |  | **Cyst** | **Single oocytes** | **Follicles** | **Total** |
| --- | --- | --- | --- | --- | --- |
| **WT (N=8)** | # | 1456 ± 203.6^a^ | 2466 ± 323.9^ab^ | 324.3 ± 46.8 | 4246 ± 416.8^ab^ |
|  | % | 34.3 ± 4.1 | 57.9 ± 3.7 | 7.8 ± 1.1 | - |
| ***Chk2*^-/-^ (N=5)** | # | 1875 ± 327.7^b^ | 3929 ± 283.2^bcd^ | 550.8 ± 119.8^ab^ | 6354 ± 457.2^acd^ |
|  | % | 28.9 ± 4.6 | 62.7 ± 5.5 | 8.3 ± 1.3 | - |
| ***Spo11*^-/-^ (N=4)** | # | 300.6 ± 22.3^abc^ | 1116.9 ± 128.3^ac^ | 183.5 ± 20.7^b^ | 1600 ± 132.2^bce^ |
|  | % | 19 ± 1.5 | 69.2 ± 3 | 11.8 ± 1.7 | - |
| ***Spo11^-/-^ Chk2^-/-^* (N=4)** | # | 1441 ± 228.1^c^ | 1680 ± 200.8^d^ | 165.8 ± 47.6^a^ | 3287 ± 453.7^de^ |
|  | % | 43.5 ± 1.1 | 51.5 ± 1.6 | 5 ± 1 | - |
| The numbers express the average ± SEM.  N indicates the number of oocytes counted.  a-e represents the statistical difference between the four genotypes (T-test). | | | | | |
