# Supplementary material for: The DNA damage response is required for oocyte cyst breakdown and follicle formation in mice
Source: PLoS Genet. 2020 Nov 18;16(11):e1009067. doi: 10.1371/journal.pgen.1009067 (PMC7710113; doi:10.1371/journal.pgen.1009067)
Supplement: S5 Table — (DOCX) [file pgen.1009067.s009.docx]

| **Genotype** |  | **Cyst** | **Single oocytes** | **Follicles** | **Total** |
| --- | --- | --- | --- | --- | --- |
| **DMSO (N=9)** | # | 94.6 ± 25.7^a^ | 559.8 ± 46.7^a^ | 824.8 ± 67.6 | 1479 ± 124.4^a^ |
|  | % | 5.9 ± 1.2^b^ | 38.1 ± 1.5^bc^ | 56 ± 1.9^b^ | - |
| **Chk1i 1 μM (N=3)** | # | 116.8 ± 48.6 | 467.3 ± 187.5 | 848.5 ± 168.7 | 1433 ± 401.3 |
|  | % | 7.4 ± 2.2 | 30.3 ± 4.1^b^ | 62.3 ± 5.8 | - |
| **Chk1i 5 μM (N=6)** | # | 320.6 ± 73.4^a^ | 1033 ± 134.2^a^ | 693.8 ± 95.5 | 2047 ± 260.5^a^ |
|  | % | 14.9 ± 1.9^b^ | 50.6 ± 2.6^c^ | 34.4 ± 3.7^b^ | - |
| The numbers express the average ± SEM.  N indicates the number of oocytes counted.  a-c represents the statistical difference between the three conditions (T-test). | | | | | |
