# Supplementary material for: The DNA damage response is required for oocyte cyst breakdown and follicle formation in mice
Source: PLoS Genet. 2020 Nov 18;16(11):e1009067. doi: 10.1371/journal.pgen.1009067 (PMC7710113; doi:10.1371/journal.pgen.1009067)
Supplement: S6 Table — (DOCX) [file pgen.1009067.s010.docx]

| **Day** | **Genotype** |  | **Cyst** | **Single oocytes** | **Follicles** | **Total** |
| --- | --- | --- | --- | --- | --- | --- |
| **Water** | **WT (N=7)** | # | 496 ± 34.2* | 3268 ± 303.6* | 54.4 ± 11 | 3818 ± 299*^a^ |
|  |  | % | 13.5 ± 1.4 | 85 ± 1.7 | 1.6 ± 0.4 | - |
|  | ***Chk2*^-/-^ (N=6)** | # | 901.2 ± 28.8* | 6207 ± 423.4* | 68.7 ± 12.1 | 7177 ± 454.5*^c^ |
|  |  | % | 12.7 ± 0.6 | 86.4 ± 0.5 | 0.9 ± 0.1 | - |
| **AZT 5 mg/kg** | **WT (N=6)** | # | 495.7 ± 64.3 | 2704 ± 256.8 | 14.7 ± 6.2 | 3215 ± 249.8^b^ |
|  |  | % | 16 ± 2.6 | 83.6 ± 2.5 | 0.4 ± 0.2 | - |
|  | ***Chk2*^-/-^ (N=4)** | # | 867.8 ± 290.6 | 5641 ± 1654 | 58.5 ± 25.2 | 6567 ± 1964 |
|  |  | % | 12.8 ± 1.3 | 86.5 ± 1.3 | 0.8 ± 0.2 |  |
| **AZT 15 mg/kg** | **WT (N=3)** | # | 724.7 ± 121.3 | 4685 ± 197.3 | 82 ± 10* | 5492 ± 206.3^abc^ |
|  |  | % | 13.2 ± 2.1 | 85.3 ± 2.1 | 1.5 ± 0.2* | - |
|  | ***Chk2*^-/-^ (N=6)** | # | 844.3 ± 50 | 6537 ± 563.2 | 48.7 ± 6.4* | 7430 ± 599.2 |
|  |  | % | 11.5 ± 0.6 | 87.8 ± 0.6 | 0.7 ± 0.1* | - |
| The numbers express the average ± SEM.  N indicates the number of oocytes counted.  *represents the statistical difference between the two genotypes (T-test). | | | | | | |
